# Comparison of Surgical Outcomes of Breast‐Conserving Surgery Performed Under Local and General Anesthesia

**DOI:** 10.1111/ans.70207

**Published:** 2025-07-02

**Authors:** Sivakanthan Thiviya, Ahmed Buraq, Agrawal Amit

**Affiliations:** ^1^ School of Clinical Medicine University of Cambridge Cambridge UK; ^2^ Harvard Medical School Boston Massachusetts USA; ^3^ Dana‐Farber Cancer Institute Boston Massachusetts USA; ^4^ Breast Unit Cambridge University Hospitals Cambridge UK

**Keywords:** breast cancer, breast‐conserving surgery, comparative study, general anesthesia, local anesthesia, oncoplastic surgery, outcomes, surgical outcomes

## Abstract

**Background:**

Surgical resection remains the cornerstone of early breast cancer treatment. However, older patients with multiple co‐morbidities may be unsuitable for general anesthesia (GA). The COVID‐19 pandemic necessitated some surgeries under local anesthesia (LA), especially those without an option for anti‐hormonal bridging therapy. This retrospective study compares the outcomes of breast‐conserving surgery (BCS) under LA and GA.

**Methods:**

Thirty‐one consecutive patients under LA (April 2018–March 2022) were compared with 31 patients under GA. The primary outcomes included the length of hospital stay, postoperative complications within 1 month (including wound infections and seromas needing ≥ 3 aspirations), margin positivity, and re‐operation rates. Statistical analysis was performed using R‐4.2.2 with significance set at *p* < 0.05.

**Results:**

Five LA cases were performed 2 years before and 26 cases after the March 2020 UK lockdown. Between the LA and GA cohorts, there was no significant difference in median ages (LA: 73 years, IQR, 82.5–69 vs. GA: 72, IQR, 82–69; *p* = 0.760) and median tumor size (LA: 16.6 mm, IQR, 21.5–12.25 vs. GA: 23.1, IQR, 30.25–14; *p* = 0.081). Three LA patients underwent Sentinel node biopsy versus 28 GA. No LA patients underwent axillary node clearance versus one GA patient. Hospital stay was significantly shorter in LA versus GA (0 vs. 10, *p* < 0.001). There were no significant differences in postoperative complications (LA: 5 vs. GA: 4, *p* = 1), positive resection margins (LA: 10 vs. GA: 18, *p* = 0.074) and re‐operation rates (LA: 1 vs. GA: 5; *p* = 0.08).

**Conclusions:**

BCS under LA increased fivefold following the pandemic. Outcomes under LA were comparable to GA, providing a viable alternative approach for those unfit for GA, thus optimizing healthcare resources.

## Graphical abstract

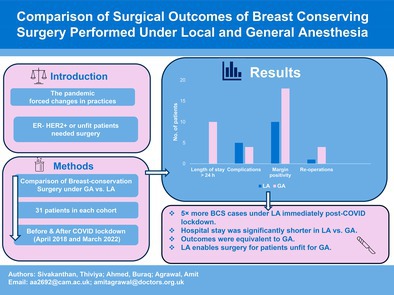

## 1 Background

Surgical resection remains the primary treatment for early breast cancer. However, a subset of older patients with multiple comorbidities may face a higher‐risk when undergoing general anesthesia (GA), prompting the need to consider alternative strategies. These alternative management approaches are guided by tumor biology. Estrogen receptor (ER) positive cancers, which are more prevalent in older individuals [1], have the option of primary endocrine therapy [2]. However, for patients with ER‐negative breast cancer, such alternative anti‐estrogen therapies are not available, necessitating the acceptance of higher‐risk anesthesia.

The COVID‐19 pandemic brought about unprecedented challenges for healthcare systems worldwide, requiring a re‐evaluation of conventional practices. Factors such as reduced bed capacity for overnight hospital stays, concerns regarding extended hospital stays with the potential for in‐hospital COVID‐19 infection, and the risks associated with aerosol‐generating procedures during GA induction and reversal all contributed to this shift [3].

In the context of the pandemic, surgery for ER‐positive breast cancer was intentionally postponed, and instead, bridging endocrine therapy was utilized as a temporary solution [4]. However, the absence of such alternatives due to capacity limitations led to a significant shift towards performing surgeries under local anesthesia (LA) for ER‐negative breast cancer or when endocrine therapy was not tolerated in ER‐positive cases. This strategic transition from GA to LA reduced the risks associated with GA and aerosol‐generating procedures [5].

Observing the evolution of our practice has prompted us to assess these developing surgical techniques and their impacts on patient outcomes. In this context, we present a comparative analysis of surgical results following breast conserving surgery (BCS) performed under LA and GA.

## 2 Methods

### 2.1 Study Design

Following institutional approval (PRN10751), a retrospective analysis of patient data from the hospital's fully electronic database was conducted. This process included ethical approval in accordance with the latest Declaration of Helsinki. Patients who underwent BCS for breast cancer at our centre between April 2018 and March 2022 were reviewed.

The inclusion criteria were:

- Female breast cancer
- Patients aged over 18
- Treated with BCS
- Node‐negative
- Under GA or LA

The exclusion criteria were breast cancer patients not suitable for or declining BCS.

We wanted to ascertain the practice for 2 years before and after the declaration of the COVID‐19 lockdown in the UK. We identified 31 consecutive patients who underwent BCS under LA and compared their outcomes with those of 31 randomly selected patients who had BCS under GA; therefore, they were not matched variables.

The LA agent utilized was a 50:50 combination of 10–20 mL of 1% Lignocaine (as a rapid onset drug), with or without epinephrine (1:200 000), and 10–30 mL of 0.25%–0.5% L‐Bupivacaine (slower onset but longer acting), also with or without epinephrine (1:200 000), administered at clinically approved doses. A larger volume can be obtained by diluting 50:50 with 0.9% normal saline to remain within the maximum dose based on body weight. To buffer the acidity of Lignocaine, 8.4% sodium bicarbonate may be added to neutralize the pH.

Using a fine hypodermic needle, the mixture was injected along the intended incision line and then surrounding the tumor in a ring‐block fashion. A few minutes later, the patient was asked about any pain sensation by gently touching and then pinching with toothed forceps (such as Adson's). Once the areas were insensitive, a deeper injection was made with longer hypodermic needles. A few minutes later, surgery proceeded. Once it was determined that the maximum depth of LA was reached, further LA infiltration was done for deeper layers of radial margins and the posterior layer. At any time, if the patient complained of pain, dissection was stopped, and more LA was infiltrated. A few minutes later, surgery proceeded. Once surgery was complete, any remaining LA was infiltrated into the walls of the cavity before cavity and skin closure.

The large majority of our breast patients have total intravenous anesthesia (TIVA) as a GA technique that reduces many of the GA side‐effects; however, it is not zero, as in LA. Most breast patients are planned as a day case following published UK day case criteria [6].

The primary outcomes were:

- Length of hospital stay.
- Post‐operative complications within 30 days of surgery as per the NHS Episode Statistics. Complications included wound infections, dehiscence, and seromas needing three or more aspirations.
- Incidence of margin positivity.
- Rate of re‐operation.

To evaluate the differences between the two groups (GA vs. LA), statistical analyses were conducted using R‐4.2.2. A two‐tailed test was used when data was normally distributed, whereas Mann–Whitney U, Chi‐Squared tests, and Fisher's exact tests were used when it was not. *p* values below 0.05 signified statistical significance.

## 3 Results

The number of BCS cases carried out under LA after the pandemic increased by more than 5 times. Five cases were performed under LA 2 years before the first UK lockdown (March 2020), whereas 26 cases were conducted in the following 2 years.

**FIGURE 1:**
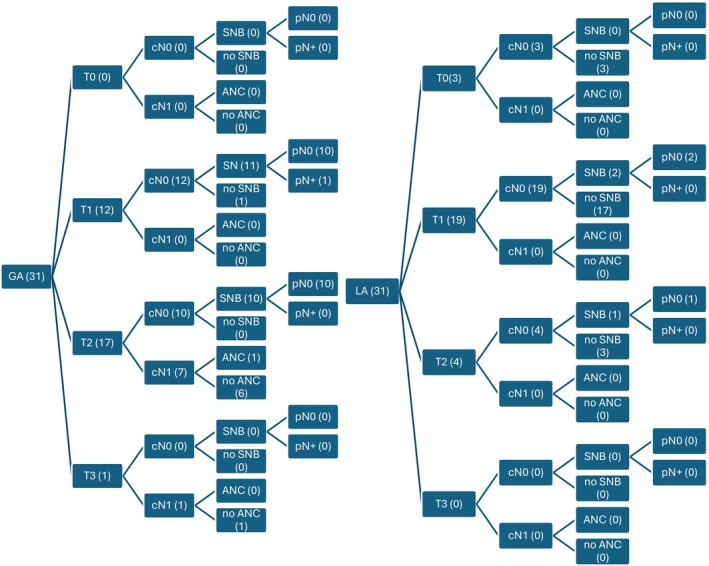
A flow chart of the tumor and nodal stage stratification. cN0, clinically node‐negative; cN1, clinically node‐positive; GA, general anesthesia; LA, local anesthesia; N/A, not applicable; pN0, node‐negative; pN1, node‐positive.

The median age was 73 years (IQR, 82.5–69) in the LA group and 72 years (IQR, 82–69) in the GA group (*p* = 0.76). The median total tumor size in LA was 16.6 mm (IQR, 21.5–12.25) while in GA it was 23.1 mm (IQR, 30.25–14) (*p* = 0.081). Figure 1 illustrates the TNM staging for both cohorts.

Sentinel node biopsy (SNB) was performed on 3 cN0 patients in the LA group and 28 in the GA group. No patients in the LA group underwent axillary node clearance (ANC) though one SNB positive underwent axillary radiotherapy. One GA patient underwent ANC with one of 11 nodes positive.

**FIGURE 2:**
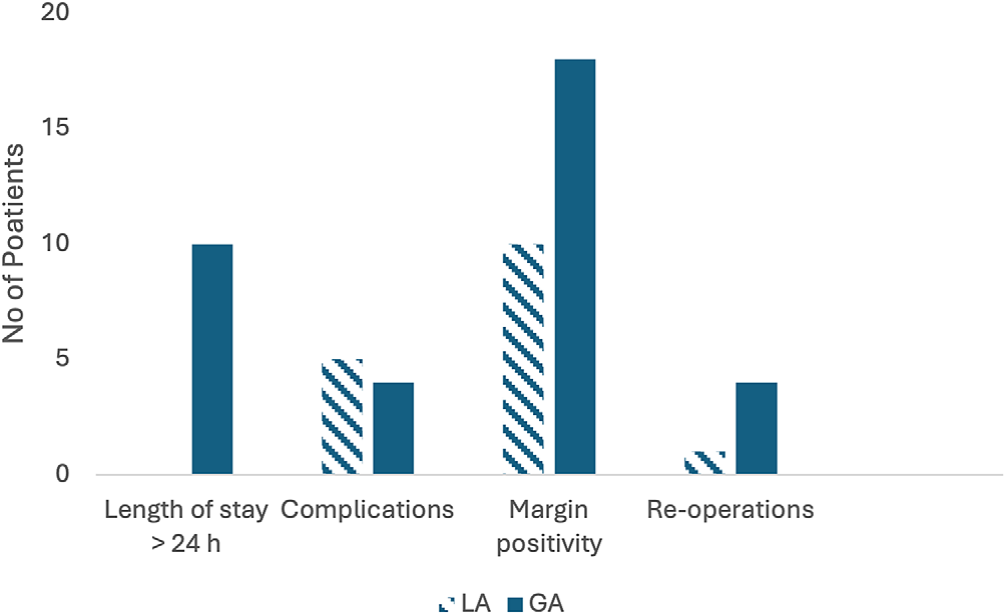
A graph showing the comparative outcomes between the LA and the GA patients who underwent BCS: 24‐h hospital stay, breast site complications in 30 days post‐operatively, positive margins, and re‐operations. BCS, breast conservation surgery; GA, general anesthesia; LA, local anesthesia.

Figure 2 shows the comparative outcomes of the LA and GA patients who underwent BCS: a 24‐h hospital stay, complications in 30 days postoperatively, positive margins, and re‐operations.

The median time for operation was significantly shorter under LA than GA (LA: 45.5 min, IQR, 31–53 vs. GA: 61, IQR, 53–70.5; *p* = 0.0038).

There was a statistically significant difference (*p* < 0.001) in the length of hospital stay between the two groups of patients. No patients in the LA group remained in the hospital for more than 24 h, whereas 10 out of 31 patients in the GA group had a hospital stay longer than 24 h (maximum 29 h). The reasons for this were unclear, although it was likely due to longer post‐operative recovery, mobility, and social factors.

There was no significant difference in the rate of 30‐day post‐operative complications between the two groups (LA: 5 vs. GA: 4; *p* = 1). Four patients in the LA group experienced wound infections that were managed with antibiotics and one haematoma (10 mls aspirated) without the need for re‐admission or in‐theater intervention. Of the four patients in the GA group, three had similar wound infections managed with antibiotics without requiring re‐admission or in‐theater intervention. The remaining patient had infected fat necrosis, which was managed non‐operatively with antibiotics.

Since axillary surgery as a second site can cause patient complications (one reason for axillary de‐escalation besides oncological needs), we determined each site's complications to differentiate complications from the breast versus the axilla. Statistically, it was impossible to compare axillary outcomes between LA and GA since the LA group had only 3 SNB. Among those who did not receive SNB, the LA group included 5 TNBC, 14 ER+/PR+, and 4 ER+/PR− cases, with no HER2+ cases; the GA group included 2 ER+/PR+/HER2− cases. Both LA and GA axilla had no complications.

Table 1 compares margin positivity and margin widths between the two cohorts. The margin positivity rate did not significantly differ between the two groups (LA: 10 vs. GA: 18, *p* = 0.074).

**TABLE 1:** Comparing margin positivity and margin widths between the two cohorts (BCS performed under LA and GA).

	Margin positivity	DCIS reaching margins	Invasive tumor reaching margins	Re‐operation	Mean superior margin distance (mm)	Mean medial margin distance (mm)	Mean inferior margin distance (mm)	Mean lateral margin distance (mm)
LA	10	5	5	1 re‐excision	8.73 (IQR, 15.0–5.0)	2.74 (IQR, 4.8–1.8)	4.72 (IQR, 6.0−3.0)	7.00 (IQR, 8.5−5.8)
GA	18	6	12	3 re‐excisions, 1 Mastectomy	7.49 (IQR, 13.5−4)	3.23 (IQR, 12.0−2.8)	1.11 (IQR, 16.3−2.0)	2.99 (IQR, 15.5−4.0)

Abbreviations: BCS, breast conservation surgery; GA, general anesthesia; IQR, inter‐quartile range; LA, local anesthesia.

One patient in the LA group had a further operation (margin re‐excision), while five patients in the GA group had further surgery (four patients had margin re‐excision, and one had a completion mastectomy). The re‐operation rate between the two groups was not significantly different (LA, 1; GA, 5; *p* = 0.086). Table 2 shows the tumor biology distribution, and Table 3 shows the treatment options in margin‐positive patients who did not undergo re‐excision surgery.

**TABLE 2:** Tumor biology distribution.

	TNBC	ER−HER2+	ER + HER2+	ER + HER2−	Others
LA	7	0	1	20	2 high‐grade DCIS 1 Metastatic leiomyosarcoma
GA	2	0	1	28	

Abbreviations: DCIS, ductal carcinoma in situ; ER, estrogen receptor; GA, general anesthesia; HER2, human epidermal growth factor receptor 2; LA, local anesthesia; TNBC, triple negative breast cancer.

**TABLE 3:** Treatment in patients with positive margins that did not undergo re‐excision.

Category	LA (*n* = 9)	GA (*n* = 14)	*p*
Adjuvant hormone therapy (HT)			1.00
Letrozole	22.2% (2/9)	71.4% (10/14)	0.036
Tamoxifen	0% (0/9)	14.3% (2/14)	0.50
Adjuvant bisphosphonate	11.1% (1/9)	7.1% (1/14)	1.00
Radiotherapy (RT)	55.6% (5/9)	100% (14/14)	0.014

Abbreviations: GA, general anesthesia; HT, hormone therapy; LA, local anesthesia; RT, radiotherapy.

The median follow‐up was not different in LA versus GA (38.5, IQR, 18–45 vs. 45, IQR, 35–49.5 months; *p* = 0.17). Local recurrence was LA: 3.6% and GA: 0% (*p* = 1), regional (axilla or around) was 0% for both cohorts, and systemic recurrence was 16.1% and 3.2% respectively (*p* = 0.197).

## 4 Discussion

The comparison of surgical outcomes for BCS performed under LA and GA is crucial, particularly in the context of increasingly limited healthcare resources (further exacerbated by the COVID‐19 pandemic). This study aimed to evaluate differences in patient outcomes and oncological efficacy.

The LA group experienced significantly shorter hospital stays than the GA group, which, while not unexpected, highlights the potential benefits of LA in rapid recovery. Future dedicated lists for BCS performed under LA will alleviate the strain on current healthcare resources. Although we did not conduct an economic analysis, it is evident that there will be substantial cost savings due to reduced anesthetic staffing, resource usage, and length of stay.

The incidence of post‐operative breast site complications within 30 days was low in both groups (LA = 5, GA = 4), with no statistically significant difference. In our data, with a 1mm margin clearance policy, margin positivity in the GA group was numerically greater than that of the LA group (*p* = 0.074), possibly due to more T2/T3 tumors in the former group. Studies with larger sample sizes may offer further clarity. LA can facilitate BCS in more patients, especially those unfit for or reluctant to undergo GA [7, 8].

Both LA and GA are associated with risks. The risks of GA are well documented and discussed pre‐operatively [9], including respiratory (atelectasis, laryngospasm), cardiovascular (hypotension, cardiac ischaemia), and neurological risks (pain or direct injury). Although rare, systemic effects have been reported with LA, such as anaphylaxis and methemoglobinemia [7]. Careful consideration of the individual patient's risks during a pre‐operative anesthetic assessment clinic and patient preferences is essential for the final decision regarding the type of anesthesia.

Performing surgery under LA may offer additional peri‐operative benefits. Karanlik et al. [7] using patient postoperative questionnaires, discovered that patients undergoing BCS under GA experienced significantly higher nausea scores (though much less with TIVA) than those under LA. This is self‐evident given the type of anesthesia; notably, they reported no significant difference in postoperative pain scores between the two groups, suggesting that LA can provide adequate pain control and may result in fewer side effects than GA. Intra‐operatively, the pain relief from LA showed no statistically significant difference compared to other techniques, including GA [10].

Margin positivity and re‐operation rates are crucial determinants of surgical oncological success. Although the rates of positive resection margins and subsequent re‐operations were numerically higher in the GA group, the differences did not reach statistical significance. This difference may be due to the inherent fitness of the GA patients for re‐operation under GA, whilst there was either acceptance of margins for LA patients or reluctance by the patients, though per se, re‐operation would be possible under LA. These findings suggest that the anesthetic modality may not significantly influence such outcomes in BCS, as noted in other studies [5, 11]. One RCT comparing peri‐tumoural lignocaine to none (with surgery under GA) reported that the lignocaine cohort significantly improved disease‐free and overall survival. Our practice involves routinely injecting LA into the wound for postoperative pain control (if not always around the tumor) during surgery under GA.

In the LA group, most patients did not undergo SNB (following multidisciplinary team approval) as axillary surgery under LA presents greater challenges and potential pain due to axillary nerve sensitivity. Consequently, this created an inherent selection bias against SNB. Most tumors had favorable prognoses or were deemed unsuitable for GA, irrespective of tumor biology. Although not the primary focus of the paper, it is worth noting that two recently reported RCTs [the Sound trial [12] and the InSema trial [13]] indicated that in T1–T2cN0 breast cancer, axillary surgery can be omitted without compromising at least 5‐year oncological outcomes compared to the SNB arm. These findings enhance the opportunities for surgery under LA, as both surgeons and patients may feel more comfortable undertaking wide local excisions without SNB under LA. Consequently, even fitter T1–T2N0 patients could opt for BCS under LA. The non‐GA approach could also be extended to those requiring axillary surgery or mastectomy, potentially incorporating additional pectoral or para‐vertebral blocks with or without sedation.

Although we encourage oncoplastic breast surgery regardless of age [14], there are likely two key limitations. First, the excision volume in oncoplastic surgery is greater due to the nature of oncoplastic techniques (and their indications); therefore, GA is likely necessary. No patient in our cohort underwent oncoplastic surgery (LA or GA) [15]. Second, oncoplastic surgery requires complex pre‐operative imaging and planning. Healthcare conditions that preclude GA will likely, if not necessarily, also preclude oncoplastic surgery [16].

The study period was short, and the sample size was small for a meaningful statistical conclusion regarding the difference in recurrences. However, we noted larger systemic recurrence in patients receiving LA compared to GA (16.1% and 3.2%, respectively; *p* = 0.197). This was a retrospective analysis; therefore, there were no associated patient‐reported outcomes measures (PROMS) data. However, it may be extrapolated that there was no undue distress or pain to the patient since all procedures were completed.

In conclusion, our study's findings strengthen the evidence regarding the applicability of LA in BCS for early breast cancer, supporting active consideration of this approach to enable surgeries that might otherwise be denied. However, the study is limited by its single‐center and small retrospective nature. While LA offers advantages such as shorter hospital stays and reduced complication rates, additional data with larger sample sizes and longer follow‐up periods are essential to substantiate specific findings, including surgical and oncological outcomes. Furthermore, qualitative research exploring patient experiences and preferences regarding anesthesia modalities could provide valuable insights into shared decision‐making and improve patient‐centred care.

## Author Contributions

**Sivakanthan Thiviya:** conceptualization, investigation, validation, visualization, methodology, data curation, writing – original draft. **Ahmed Buraq:** writing – review and editing, writing – original draft, formal analysis, data curation, resources, investigation, validation, visualization. **Agrawal Amit:** conceptualization, investigation, methodology, writing – original draft, writing – review and editing, project administration, supervision, resources.

## Disclosure

A.A. is affiliated with the University of Cambridge, UK as an Associate Professor and is supported by the NIHR Cambridge Biomedical Centre (BRC‐1215‐20014), Addenbrooke's Charitable Trust grant (900353), NIHR grant (NIHR205746), UK Govt. Office for Technology Transfer KAGF via UK Research & Innovation grant (10073066) and Cancer Research UK Primer award (EDDPMA‐Nov23/100027).

## Ethics Statement

Institutional approval (PRN10751) was gained for this clinical evaluation.

## Conflicts of Interest

The authors declare no conflicts of interest.
